# Novel Impedance‐Guided Contact Mapping Technique for the Circular Multielectrode Pulsed‐Field Ablation Catheter

**DOI:** 10.1002/ccr3.72142

**Published:** 2026-02-24

**Authors:** Kennosuke Yamashita, Yohei Kikuchi, Keita Yoshiyama, Daiki Kumazawa, Yosuke Mizuno, Kosuke Onodera

**Affiliations:** ^1^ Heart Rhythm Center, Department of Cardiovascular Medicine Sendai Kosei Hospital Miyagi Japan

**Keywords:** contact index, impedance mapping, pulsed field ablation, PulseSelect catheter, zero fluoroscopy

## Abstract

Impedance‐guided PulseSelect contact mapping (i‐Pulse) adapts EnSite X Contact Index for a circular multielectrode PFA catheter, enabling real‐time visualization of electrode–tissue contact without fluoroscopy. With ICE guidance, Δimpedance > 5% identified adequate contact, standardized tagging, and guided applications in three atrial fibrillation cases, achieving acute pulmonary vein isolation without complications.

**Abbreviations**
3D: three‐dimensional
AF: atrial fibrillation
ICE: intracardiac echocardiography
IQR: interquartile range
LA: left atrium
LAA: left atrial appendage
LIPV: left inferior pulmonary vein
LSPV: left superior pulmonary vein
PFA: pulsed field ablation
PV: pulmonary vein
RIPV: right inferior pulmonary vein
RSPV: right superior pulmonary vein
Δimpedance: percent change in impedance from the blood‐pool baseline (%)

## 1 Introduction

Pulmonary vein isolation (PVI) using pulsed field ablation (PFA) has gained widespread adoption for atrial fibrillation treatment because of its favorable safety profile and clinical efficacy, as demonstrated in trials such as the PULSED AF trial [1]. However, conventional PFA workflows still rely heavily on fluoroscopy [2], exposing patients and operators to ionizing radiation. Despite the nonthermal nature of PFA, effective lesion formation requires adequate electrode–tissue proximity [3]. Because PFA catheters typically lack contact‐force sensors, fluoroscopy is often used as an auxiliary method to confirm contact, increasing radiation exposure.

Technological advances, including 3D electroanatomical mapping systems and intracardiac echocardiography (ICE), have substantially reduced fluoroscopy by providing integrated electrical and anatomical guidance [4, 5]. However, ICE alone may be insufficient to confirm electrode contact at all sites, especially when ICE is not advanced into the left atrium because of the risk of iatrogenic atrial septal defects.

The VARIPULSE catheter (Biosense Webster) integrated with the CARTO system uses an impedance‐based Tissue Proximity Indicator for contact assessment. Recently, the EnSite X EP System (Abbott) introduced a similar impedance‐based “Contact Index” for the FARAPULSE catheter (Boston Scientific). However, this function is not directly compatible with the PulseSelect catheter (Medtronic). To address this limitation, we propose i‐Pulse (Impedance‐guided PulseSelect Contact Mapping), an adaptation of the EnSite X Contact Index concept for PulseSelect, enabling real‐time visualization of tissue contact based on impedance dynamics without fluoroscopy.

## 2 Case History/Examination

We report three consecutive patients with atrial fibrillation (age 57–71 years; 1 man) who underwent pulmonary vein isolation using the PulseSelect pulsed field ablation catheter with EnSite X and intracardiac echocardiography guidance. To minimize procedural interruptions from patient movement or reflexive coughing during PFA, general anesthesia was induced with propofol and rocuronium. Two venous access sites were obtained under ultrasound guidance: an 8.5‐Fr SL0 sheath (Abbott) for transseptal puncture and a 10‐Fr, 30‐cm sheath for ICE using the ViewFlex Xtra catheter (Abbott). A radiofrequency needle (Sepnee; Kaneka Medix) was advanced through the SL0 sheath to the fossa ovalis, and successful transseptal puncture was confirmed by ICE. The SL0 sheath was then exchanged for a FlexCath Advance sheath (Medtronic).

**FIGURE 1:**
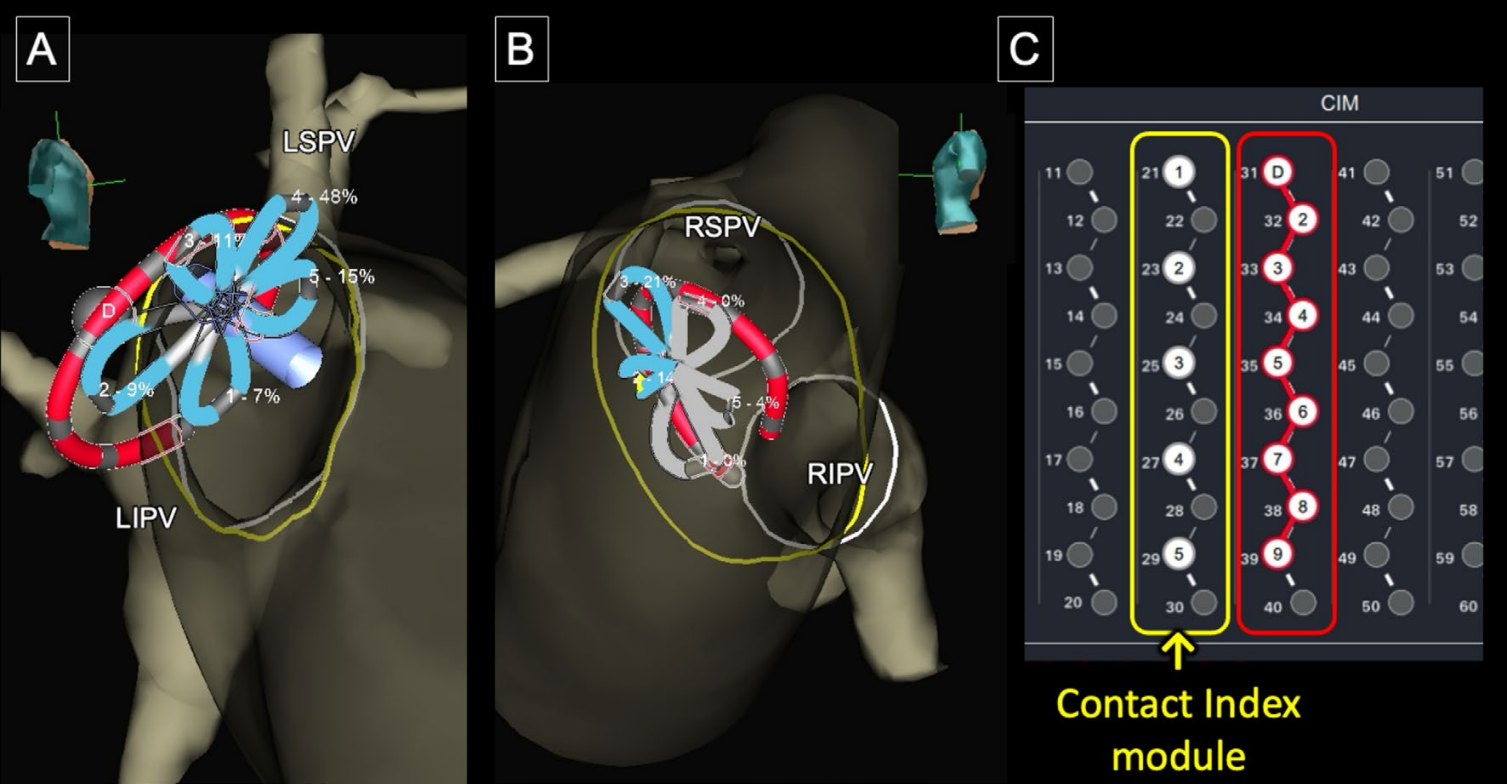
(A) PulseSelect electrodes 1, 3, 5, 7, and 9 mapped to FARAPULSE E1–E5 for Contact Index; blue indicates Δimpedance > 5%. (B) Example in the RSPV showing contact/non‐contact electrodes. (C) Jumper‐cable connections for Contact Index (yellow) and ring visualization (red).

Left atrial geometry and magnetic field calibration were performed using the Advisor HD Grid X catheter (Abbott) on the EnSite X system in Voxel mode during sinus rhythm. To facilitate guidewire visualization, the proximal end of the wire was electrically connected to the mapping system. Although the PulseSelect catheter contains nine electrodes, the EnSite X Contact Index (version 3.1.1) supports impedance‐based contact assessment for only five electrodes, as it was originally developed for the FARAPULSE system. Accordingly, electrodes 1, 3, 5, 7, and 9 of the PulseSelect catheter were mapped to the five‐electrode FARAPULSE configuration (Figure 1A,B).

The Contact Index can be measured in two modes: bipolar and tripolar. Bipolar mode calculates impedance between adjacent electrodes on the FARAPULSE configuration, whereas tripolar mode calculates impedance between a central electrode and three virtual surrounding electrodes. Either mode can be applied to the PulseSelect catheter; however, we adopted bipolar mode, which measures impedance between adjacent electrodes and enables local impedance assessment. Validation was performed using bipolar‐mode Contact Index, with the jumper‐cable connections shown in Figure 1C.

**FIGURE 2:**
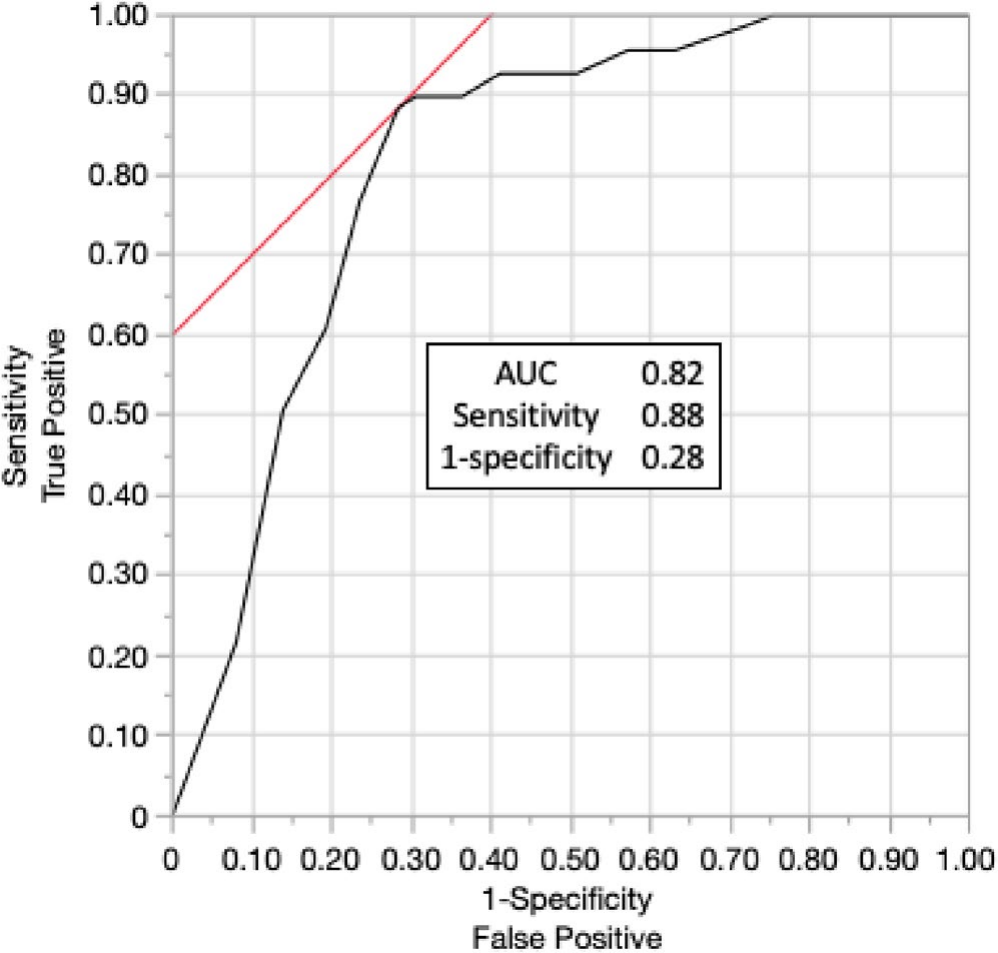
ROC analysis of Δimpedance (%) in the LA body for ICE‐defined contact vs. non‐contact; Δimpedance 5% maximized the Youden index.

The core of the i‐Pulse workflow is the application of the EnSite X Contact Index function. Five of the nine electrodes on the PulseSelect catheter (electrodes 1, 3, 5, 7, and 9) were connected to the Contact Index module to measure local bipolar impedance between adjacent electrodes. During sinus rhythm, blood‐pool impedance was first obtained with the PulseSelect catheter. After reconstructing left atrial (LA) geometry and confirming the anatomy, electrode 5 was advanced under ICE guidance and brought into gentle contact with the LA body, where local bipolar voltage and peak frequency were recorded. Sites with ICE‐confirmed wall apposition and bipolar amplitude ≥ 0.5 mV were classified as contact points; all others were classified as non‐contact points. Because impedance may rise within the pulmonary veins irrespective of true tissue contact, validation was performed exclusively using points acquired in normal‐voltage areas (≥ 0.5 mV) of the LA body under continuous ICE monitoring. In a preliminary validation phase of 10 consecutive cases, 256 points were acquired. Receiver‐operating characteristic analysis demonstrated that the percentage change in impedance from the blood‐pool baseline (Δimpedance, %) discriminated contact from non‐contact sites (AUC 0.82), and the Youden index was maximal at Δimpedance = 5% (sensitivity 0.88, 1–specificity 0.28; Figure 2). On this basis, a cutoff of Δimpedance > 5% was adopted as an objective indicator of adequate tissue contact and ablation suitability.

For Contact Index display on EnSite X (version 3.1.1), PulseSelect electrodes 1, 3, 5, 7, and 9 were mapped to the five‐electrode FARAPULSE configuration (E1–E5) via jumper cables. The system then automatically reported the percent change in impedance from the blood‐pool baseline (Δimpedance, %), which was visualized on the circular catheter and used for tagging and application guidance.

Local impedance values differ between the pulmonary veins and the LA body, with PV values tending to be higher even without contact. Therefore, only LA body measurements were analyzed. When the Contact Index cutoff was set at 5%, it correlated well with both bipolar voltage and ICE‐based assessments; accordingly, this cutoff was used to guide ablation.

**FIGURE 3:**
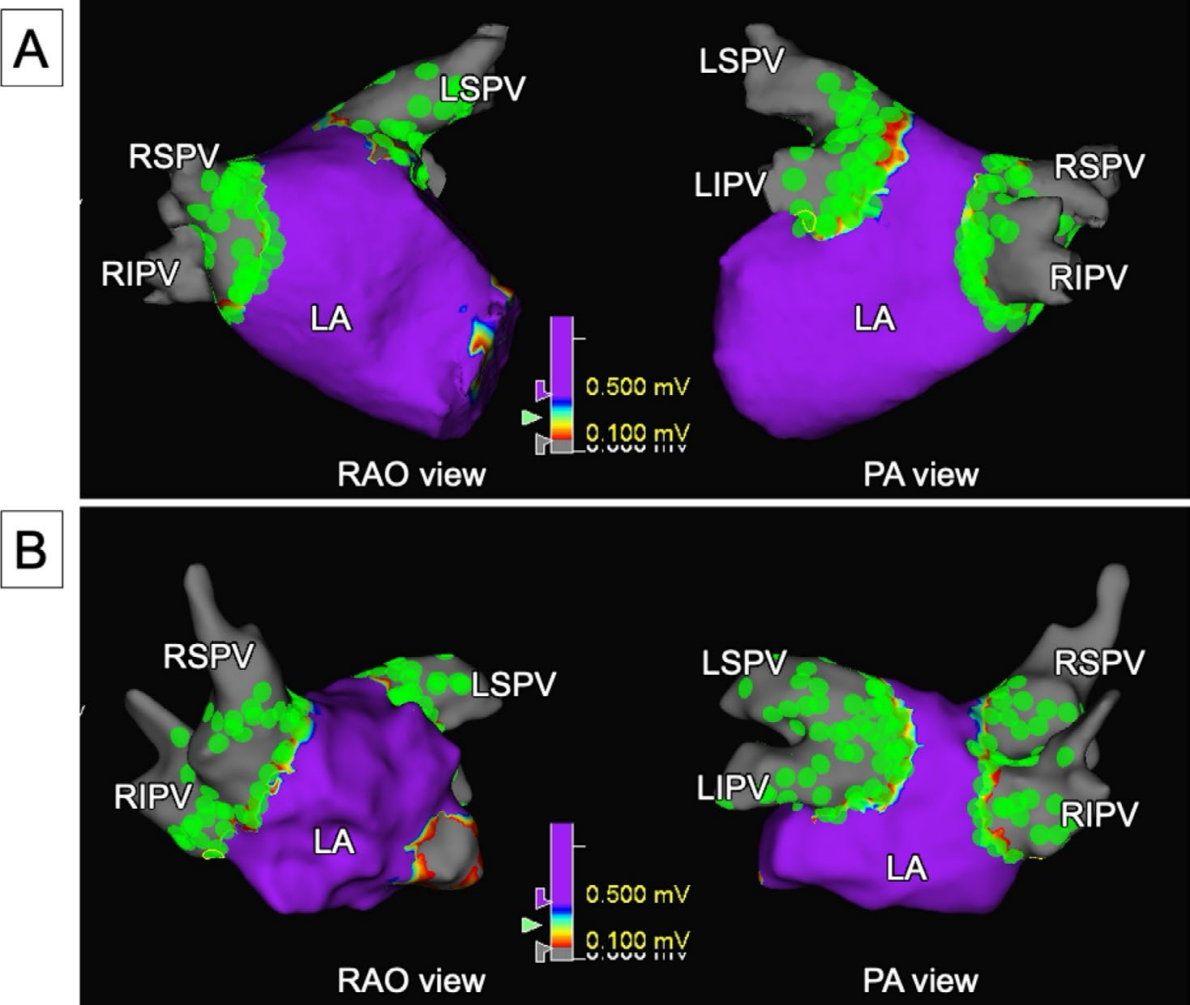
Post‐ablation voltage maps showing representative examples from two cases. (A) (Case 1): Voltage map corresponding to Figure 1, demonstrating complete PVI at sites tagged in green (Δimpedance > 5%, tag diameter 5 mm). (B) (Case 3): Another representative case with contiguous tags and no acute gaps, indicating a complete lesion set.

Minimum required applications were delivered at each site to ensure adequate lesion coverage (Figure 3). Additional applications were placed to achieve complete circumferential coverage around the pulmonary veins based on the tags. After lesion delivery, post‐ablation voltage mapping using the HD Grid X catheter and omnipolar technology confirmed the absence of residual PV potentials and the presence of entrance and exit block.

Supplementary Video 1 (Video S1) presents the first procedure (Case 1). Lesions were created based on a computed‐tomography–fused geometry reconstructed using the HD Grid X catheter. Before ablation, anatomical landmarks were tagged: PV ostia were marked with white lines and PV antra with yellow lines. Isolation was initiated from the right inferior pulmonary vein (RIPV). After confirming over‐the‐wire positioning and advancement into the RIPV, lesions were delivered according to the Contact Index, and corresponding tags were placed on the 3D geometry.

Because this was our first case, right‐sided PV isolation required 16 min and left‐sided PV isolation required 13 min. Nevertheless, the procedure was completed entirely without fluoroscopy.

### 2.1 Diagnosis and Patient Selection

The diagnosis was symptomatic, drug‐refractory paroxysmal or persistent atrial fibrillation confirmed by 12‐lead electrocardiograms and Holter monitoring. Differential diagnoses included atrial flutter, focal atrial tachycardia, atrioventricular nodal re‐entrant tachycardia/atrioventricular re‐entrant tachycardia, and frequent atrial ectopy; these were excluded during the electrophysiological study. This report includes three consecutive patients who underwent first‐time PVI using the PulseSelect catheter under EnSite X and intracardiac echocardiography guidance at our institution.

## 3 Outcome and Follow‐Up

A summary of all three procedures is provided in Table 1. Data are presented as medians with interquartile ranges (IQR). The times required for left and right PV isolation were 10.0 (IQR 10.0–12.0) minutes and 13.0 (IQR 12.0–14.5) minutes, respectively. Premapping and postmapping times were 7.8 (IQR 7.6–9.0) minutes and 7.0 (IQR 6.6–8.8) minutes, with a left atrial dwell time of 43.0 (IQR 41.0–45.5) minutes. All procedures were performed without fluoroscopy (fluoroscopy time 0.0 min).

**TABLE 1:** Clinical characteristics and pre‐procedural medications of each case.

Case	1	2	3
Age	71	63	57
Gender	Male	Female	Female
CHA_2_DS_2_‐VAS score	2	2	2
Height, cm	165.0	156.0	159.0
Body Weight, kg	80.0	46.0	50.0
Body Mass Index, kg/m^2^	29.4	18.9	19.8
Type of Atrial Fibrillation	Persistent	Paroxysmal	Paroxysmal
Medication History (pre‐procedure)	Edoxaban, bisoprolol	Edoxaban	Edoxaban
Left Atrial Diameter (mm)	43	33	31
LV End‐Diastolic Diameter, mm	51	44	45
LV End‐Systolic Diameter, mm	38	28	27
LV Ejection Fraction, %	51	67	71
PV Anomaly	None	Left common	None
PV Diameter, min			
Left Superior PV	23	25 (common)	15
Left Inferior PV	16	N/A	19
Right Superior PV	22	18	20
Right Inferior PV	25	19	19
Number of Applications			
Left Superior PV	17	24 (common)	15
Left Inferior PV	11	N/A	11
Right Superior PV	18	10	11
Right Inferior PV	12	14	12
Total	58	48	50
Left PV Isolation Time, min	13	9	11
Right PV Isolation Time, min	16	11	13
Left Atrial dwell Time min	48	39	43
Fluoroscopy Time, min	0	0	0
Fluoroscopy Dose, mGy	0	0	0

Abbreviations: LV , left; N/A , not applicable; PV , pulmonary vein.

In Case 2, a left common PV was present. Ablation was performed in the superior and inferior branches of the common PV, followed by antral applications to complete isolation. Despite this anatomical variation, LA dwell time remained short (39 min).

Pulmonary vein isolation was achieved in all cases, and no additional ablation was required after post‐mapping. No procedural complications—including pericardial effusion, esophageal injury, phrenic nerve palsy, or anesthesia‐related events—were observed. All patients were discharged the following day without adverse events.

The i‐Pulse workflow is a practical, reproducible, fluoroscopy‐sparing method for PFA using the PulseSelect catheter. Larger prospective studies with long‐term follow‐up are warranted to confirm durability and generalizability.

## 4 Discussion

Our findings extend previous reports, such as Palmeri et al. [4], which demonstrated the feasibility of zero‐fluoroscopy PFA using the FARAPULSE catheter with the EnSite NavX system and ICE. Their workflow projected ablation tags onto a pre‐acquired geometry using impedance‐based mapping. However, applying this approach to the PulseSelect catheter is challenging because of its thinner shaft, smaller electrodes, and limited ICE visualization. PulseSelect also requires multiple rotations (e.g., 12, 3, 6, and 9 o'clock) to achieve 360° PV isolation, which can be technically demanding under ICE‐only guidance. Another limitation of relying solely on NavX mode is geometry shift due to respiratory motion, PFA‐related effects, and impedance variability. To mitigate this, we used Voxel mode, which provides magnetic field‐based navigation with higher spatial accuracy. Although PulseSelect lacks magnetic sensors and requires manual tagging, real‐time ICE visualization combined with impedance‐guided mapping compensates for these constraints.

The i‐Pulse workflow also avoids pitfalls associated with bipolar potential‐based contact assessments, such as bipolar blindness. Nishiuchi et al. [6] proposed the pre‐PFA application peak‐frequency (PPAP) map, which estimates catheter–tissue contact based on bipolar electrogram frequency before ablation. While informative, this method has limitations: (1) susceptibility to far‐field contamination and distortion from fibrotic tissue; (2) disappearance of local electrograms after a single application, making it difficult to confirm stable contact; and (3) uncertain reliability of bipolar frequency near ablation lesions. Furthermore, the PPAP map relies solely on bipolar frequency, whereas unipolar frequency—which may better reflect the electrode–tissue interface—is not currently supported.

**FIGURE 4:**
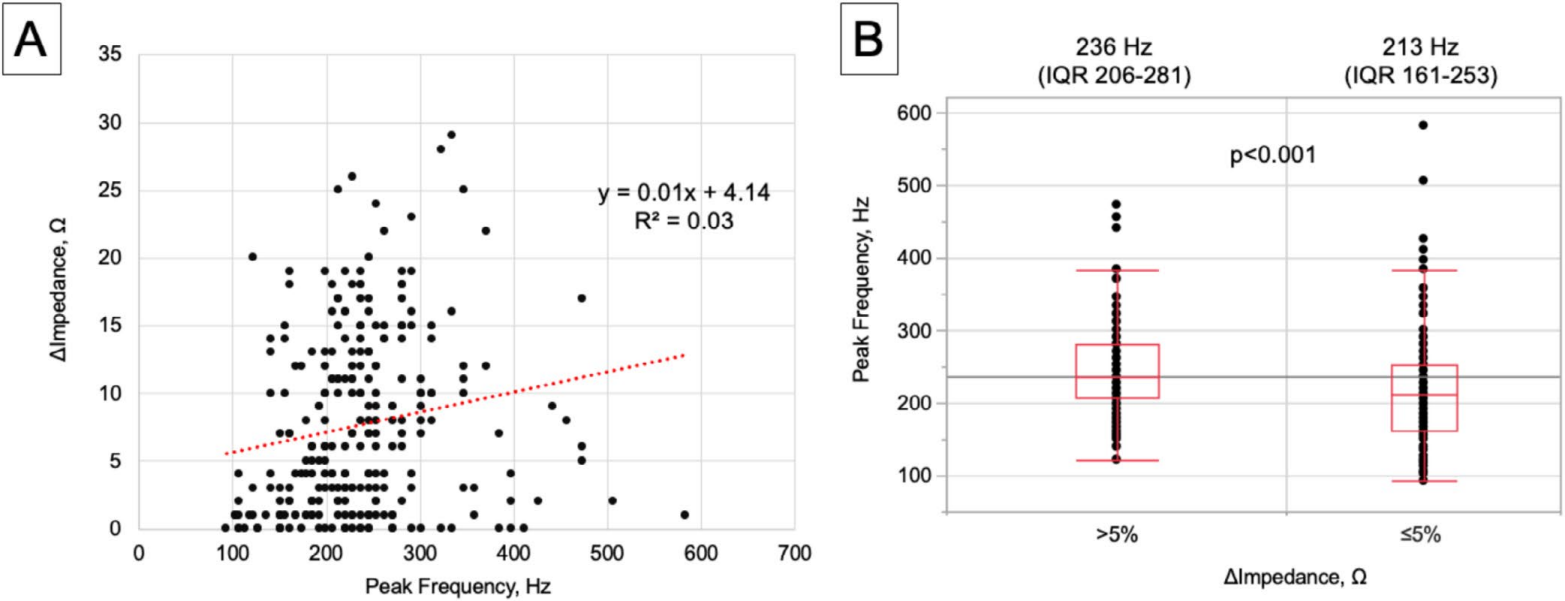
(A) Relationship between Δimpedance and peak frequency across 256 points. The regression equation is Y = 0.01 X + 4.14 (*R* ^2^ = 0.03), indicating no meaningful correlation. (B) Peak‐frequency distributions at sites with Δimpedance > 5% versus ≤ 5%. Although a statistically significant difference was observed, substantial overlap prevented determination of a practical cutoff. LA, left atrium; LAA, left atrial appendage; LIPV, left inferior pulmonary vein; LSPV, left superior pulmonary vein; PVI, pulmonary vein isolation; RIPV, right inferior pulmonary vein; RSPV, right superior pulmonary vein.

In contrast, i‐Pulse uses local impedance measured between adjacent electrodes, enabling objective, reproducible contact assessment independent of activation direction or voltage drop. Across 256 points, Δimpedance (%) showed no meaningful correlation with peak frequency (*R* ^2^ = 0.03; Figure 4A). Although peak frequency differed statistically between sites with Δimpedance > 5% and ≤ 5%, substantial overlap prevented identification of a usable cutoff (Figure 4B). These results suggest that impedance‐based contact assessment remains feasible even in regions with low peak‐frequency values.

Several limitations should be noted. First, because Contact Index was developed for FARAPULSE, only five electrodes can be monitored simultaneously when applied to PulseSelect. Second, baseline impedance may decrease during the procedure owing to intravenous fluid, anesthetic effects, or post‐ablation changes, potentially leading to underestimated contact. In such cases, ICE remains essential, and baseline impedance should be reacquired if catheter manipulation or ICE findings do not align with impedance data. Given that PulseSelect has no irrigation and the total PVI duration is short (approximately 20–30 min), the overall impact of baseline drift is likely small.

Overall, our workflow integrates EnSite X mapping and ICE to enable zero‐fluoroscopy PFA with PulseSelect. By combining impedance‐based 3D mapping with ICE, accurate catheter localization and safe energy delivery were achieved. General anesthesia further stabilized the anatomy by eliminating respiratory artifact and patient movement. Although contact verification with PulseSelect can be challenging—particularly during transitions from linear to circular configurations—ICE remains invaluable for confirming ring deployment and detecting entanglement or mechanical complications.

## Author Contributions

**Kennosuke Yamashita:** conceptualization, formal analysis, investigation, writing – original draft. **Yohei Kikuchi:** data curation. **Keita Yoshiyama:** investigation, writing – review and editing. **Daiki Kumazawa:** writing – review and editing. **Yosuke Mizuno:** writing – review and editing. **Kosuke Onodera:** writing – review and editing.

## Funding

The authors have nothing to report.

## Ethics Statement

The ethical committee of Sendai Kousei Hospital waived the requirement for obtaining ethical approval because this research was neither a clinical study nor an animal experiment.

## Consent

Written informed consent was obtained from the patient for publication of this case report and accompanying images.

## Conflicts of Interest

K.Y. received speaker honoraria and lecture fees from Abbott Medical Japan, Biosense Webster, Daiichi Sankyo, and Medtronic. The other authors declare no conflicts of interest.

## Supporting information


**Video S1:** Demonstration of the zero‐fluoroscopy i‐Pulse workflow in Case 1. The video illustrates pulmonary vein isolation starting from the right inferior pulmonary vein (RIPV). Key procedural steps include the visualization of computed‐tomography–fused 3D geometry, anatomical tagging of the PV ostia (white lines) and antra (yellow lines), and lesion delivery guided by the EnSite X Contact Index without the use of fluoroscopy.

**Data S1:** Place holder image.

## Data Availability

Raw data were generated at Sendai Kousei Hospital. Derived data supporting the findings of this study are available from the corresponding author, Kennosuke Yamashita, upon request.
