# Supplementary figures and images for: Novel Impedance‐Guided Contact Mapping Technique for the Circular Multielectrode Pulsed‐Field Ablation Catheter
Source: Clin Case Rep. 2026 Feb 24;14(3):e72142. doi: 10.1002/ccr3.72142 (PMC12932318; doi:10.1002/ccr3.72142)

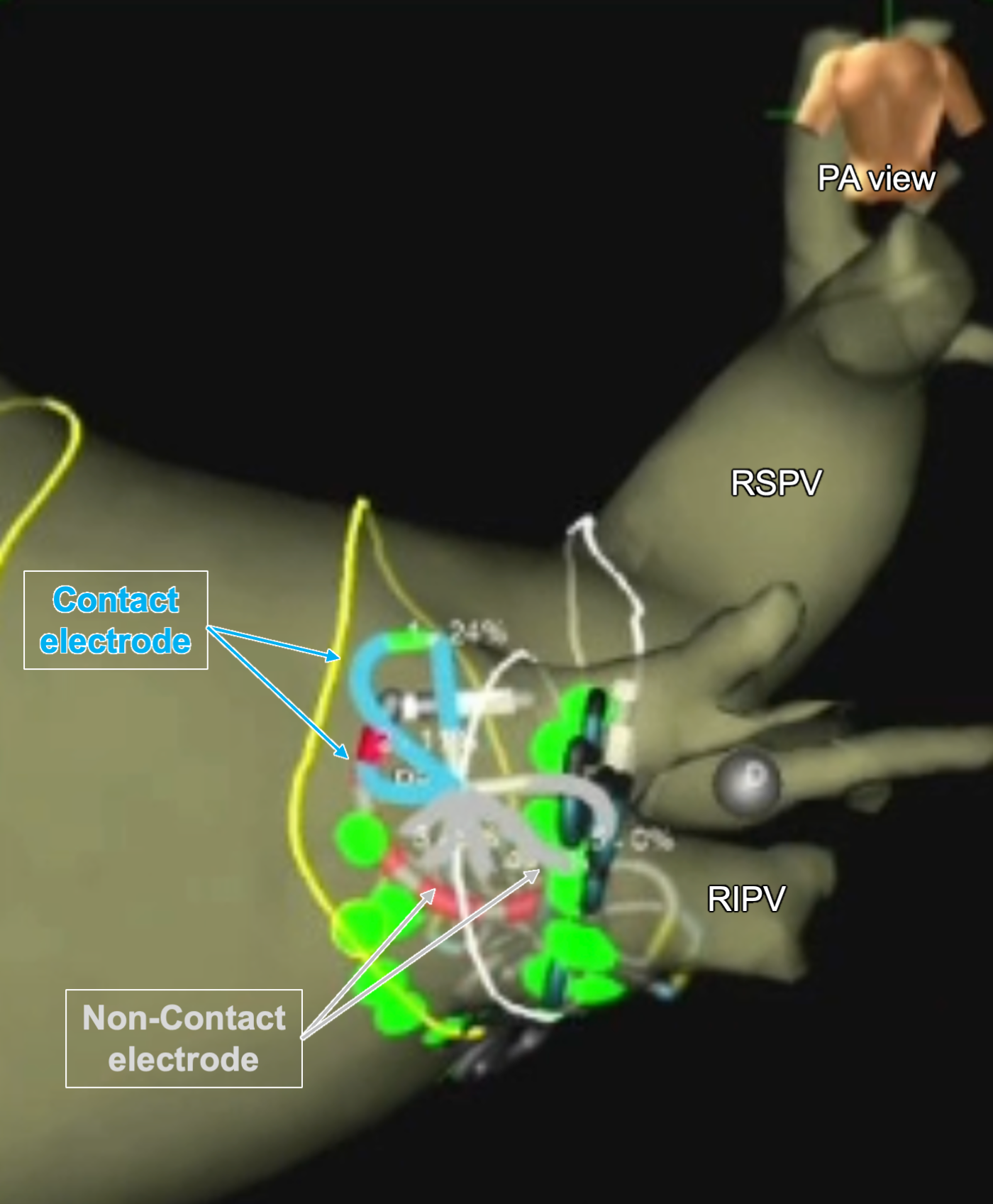

Supplement: Supplementary file 2 — Data S1: Place holder image. [file CCR3-14-e72142-s001.tiff]
